# Safety and reduction of pasteurized donor human milk waste using a 48-h post-thaw usage policy: a retrospective observational and bacteriological study

**DOI:** 10.1186/s13006-026-00877-y

**Published:** 2026-08-04

**Authors:** Hinako Yoshino, Tomomasa Terada, Katsumi Mizuno

**Affiliations:** Department of Pediatrics, Showa Medical University School of Medicine, 1-5-8 Hatanodai, Shinagawa-ku, Tokyo, Japan

**Keywords:** Donor human milk, Thawing, Human milk bank, Waste reduction, Neonatal intensive care, Microbiological safety, Breastfeeding support

## Abstract

**Background:**

Pasteurized donor human milk (PHDM) is recommended for preterm infants when the mother’s own milk is unavailable, as it reduces complications such as necrotizing enterocolitis and supports the early initiation of human milk feeding. Current guidelines in Japan require thawed PDHM to be discarded after 24 h, although emerging evidence suggests that PDHM may remain safe for up to 48 h. This study evaluated the microbiological safety of PDHM at 48 h post-thawing and estimated the amount of waste that could be potentially reduced if the allowable use period were extended.

**Methods:**

Forty PHDM samples provided by the Japan Human Milk Bank were thawed and stored at 4 °C. Aliquots were collected at 24 and 48 h post-thawing for quantitative bacterial culture. To estimate potential waste reduction, the medical records of 23 neonates who received PDHM were retrospectively reviewed. For each neonate, the actual PDHM intake, thawed volume, and discarded volume were recorded up to day 15 of life, and the expected reduction in waste under a 48-h use policy was calculated.

**Results:**

No bacterial growth was detected in any sample at 24–48 h post-thawing. Among 23 preterm infants who received PDHM, extending the allowable post-thaw usage period to 48h was estimated to save 2,051 mL of PDHM and potentially reduce discarded volume by 84.7%.

**Conclusions:**

Under routine neonatal intensive care units (NICU) conditions, PDHM remained microbiologically sterile for up to 48 h after thawing. Extending the usage period to 48 h could substantially reduce PDHM waste and help maintain a more stable supply of donor human milk in the NICU. Ensuring a stable PDHM supply may facilitate the early initiation of human milk feeding and support breastfeeding.

## Background

In 2019, the Japan Pediatric Society issued recommendations on enteral nutrition for preterm and very low birth weight infants, stating that mother’s own milk is the preferred source of nutrition and donor human milk is recommended when the mother’s milk is unavailable [1].

Pasteurized donor human milk (PDHM) is therefore widely used in neonatal intensive care units (NICU) for preterm infants when mother’s own milk is not available.

According to Vital Statistics published by the Ministry of Health, Labour and Welfare of Japan, the number of births in Japan declined markedly from approximately 1.19 million in 2000 to approximately 770,000 in 2023 [2]. In contrast, according to data published by the Children and Families Agency of Japan, the proportions of low birth weight infants (< 2500 g) and very low birth weight infants (< 1500 g) have not changed substantially [3]. Consequently, the proportion of high-risk neonates requiring admission to the NICU has remained largely unchanged over the past two decades.

In a study of 22,248 preterm infants born between 22 and 28 weeks of gestation, the most common causes of death during NICU hospitalization were immaturity and respiratory diseases, followed by necrotizing enterocolitis (10.1%) and infections (8.0%) [4]. In preterm infants, a higher proportion and prolonged administration of human milk in enteral feeding has been associated with lower incidences of necrotizing enterocolitis, bronchopulmonary dysplasia, retinopathy of prematurity, and late-onset sepsis [5]. These findings indicate that the early initiation of enteral feeding with human milk is important for preventing complications in preterm infants.

However, maternal milk supply is often insufficient during the early postnatal period in mothers of preterm infants. In such cases, PDHM is recommended as a safe alternative for human milk feeding. PDHM serves as a bridge until the mother’s own milk supply becomes sufficient and supports the early initiation and continuation of breastfeeding.

Human milk banking began in Japan in 2014, and the Japan Human Milk Bank Association was established in 2017. Currently, 114 facilities nationwide use PDHM, and demand continues to increase [6].

As PDHM is obtained solely through voluntary donations, it is a finite medical resource that must be used effectively. The existing Japanese guidelines require thawed PDHM to be used within 24 h [7], following the earlier recommendations of the Human Milk Banking Association of North America (HMBANA). Consequently, substantial volumes of thawed PDHM are discarded in NICU, particularly because PDHM is supplied in fixed-volume bottles that often exceed the daily requirements of neonates.

The HMBANA revised its guidelines in 2019 to allow the use of PDHM within 48 h of thawing [8], and several human milk banks extended the allowable usage period to 48 h.

Extending the allowable post-thaw usage period of PDHM and reducing unnecessary waste may help ensure a stable supply of PDHM, thereby supporting the early establishment of human milk feeding and potentially contributing to prolonged human milk feeding in preterm infants.

In Japan, a laboratory-based evaluation conducted at a human milk bank has demonstrated the microbiological safety of PDHM for up to 48 h after thawing [9]; however, evidence from real-world clinical settings remains limited.

This study aimed to determine whether PDHM remains microbiologically safe up to 48 h post-thaw under routine NICU conditions and to estimate the potential reduction in PDHM waste if a 48-h usage policy is implemented.

## Methods

### Study design

This study consisted of a bacteriological assessment of PDHM stored for up to 48 h after thawing and a retrospective review to estimate potential waste reduction. This study was approved by the Showa Medical University Ethics Committee (Approval No. 2714; June 21, 2021).

### Setting

The study was conducted in the NICU of Showa Medical University, a tertiary perinatal center in Tokyo that admits more than 300 infants annually, including over 20 very-low-birthweight infants (< 1500 g) who typically receive PDHM.

### PDHM samples

Individual milk samples from 40 donors, collected between May 2022 and March 2023, were used. The sample size was determined with reference to previous microbiological studies evaluating the microbiological safety of PDHM after thawing [9].

All milk samples were obtained from human milk donated to the Japan Human Milk Bank Foundation, and all donors met eligibility criteria. The donor health status was confirmed based on medical history, physical examination, and laboratory tests, in accordance with the Human milk bank utilization manual (Japan Human Milk Bank Association, 2026) [7]. These human milk samples were pasteurized using the standard procedure routinely employed by the milk bank (62.5 °C for 30 min), passed microbiological screening performed by the milk bank both before and after pasteurization prior to distribution, and were subsequently dispensed into three bottle volumes (30 mL, 80 mL, and 150 mL) for clinical use according to routine practice.

Donor milk shipped from the milk bank to our NICU was stored at − 30 °C in a freezer, consistent with routine storage conditions for expressed human milk. Thawing was performed using a warm-air circulating milk thawer located in the NICU and required approximately 60 min. After thawing, the milk was stored under routine conditions in a 4 °C refrigerator, designated for thawed human milk. Samples were thawed in five batches and subsequently divided into 24-h and 48-h analyses to minimize inter-batch variability. During the 24- and 48-h storage periods, all samples were kept at a controlled temperature of 4 °C.

To evaluate PDHM waste, we retrospectively reviewed the medical records of 23 preterm infants who received PDHM in the NICU at Showa Medical University Hospital between January 2024 and December 2024. The evaluation period was limited to the first 15 days of life, during which enteral feeding is typically established and stabilized under routine nutritional management.

### Microbiological procedures

A sterile 3 mL aliquot of each sample was submitted to a contracted clinical laboratory (BML Inc., Tokyo), where all microbiological analyses were performed. Bacterial cultures were conducted before and after pasteurization, as well as at 24-h and 48-h after thawing, in accordance with the standard culture procedures of the Human Milk Banking Association of North America (HMBANA, 2013).

From each sample, 100 µL was plated on sheep blood agar. After aerobic incubation at 35 °C for 48 h, the colony counts were measured using a Quebec colony counter (Reichert, Depew, NY, USA). The results were reported as colony-forming units per 100 µL (CFU/100 µL).

### Estimation of PDHM waste

For each infant, the volume of PDHM administered, the volume of PDHM thawed, and the volume of PDHM discarded after expiration of the post-thaw usage period were recorded. Subsequently, under a simulated 48-h post-thaw usage policy, excess PDHM that would have been discarded under the routine 24-h policy was assumed to be carried over for use on the following day. Based on this assumption, the thawed volume and discarded volume were recalculated to estimate the potential reduction in PDHM waste associated with extending the allowable post-thaw usage period to 48 h.

The attending physician determined the prescribed feeding volume for each infant. When the infant’s own mother’s milk was available, PDHM was used to supplement the remaining feeding volume. The volume of PDHM to be thawed was determined by the nursing staff. Because PDHM was supplied in standardized bottles of 30, 80, and 150 mL, the thawed volume did not always correspond to the volume of PDHM used, resulting in excess PDHM after feeding.

The study variables included infant gestational age, birth weight, the volume of PDHM administered, the volume of PDHM thawed, the volume of PDHM discarded under the routine 24-h post-thaw usage policy, and the volume of PDHM saved under the simulated 48-h policy. Data were retrospectively extracted from the electronic medical records and nursing records by one of the investigators (T.T.).

### Statistical analysis

This study was descriptive in nature. Infant characteristics and PDHM-related variables are presented for each infant, and total PDHM volumes were calculated for the 24-h and simulated 48-h post-thaw usage policies. No inferential statistical analyses were performed.

## Results

No bacterial growth was detected in any of the 40 PDHM samples cultured for 24–48 h after thawing.

For the 23 infants who required PDHM, a total of 22,480 mL of PDHM was thawed. Of this, 2,419 mL was discarded because it exceeded the allowable post-thaw usage period. If the post-thaw usage period were extended to 48 h, the projected volume of PDHM requiring thawing would be 20,440 mL, and the estimated discarded volume would be 368 mL. Thus, extending the usage period from 24 to 48 h would save 2,051 mL of PDHM, corresponding to a 84.7% reduction in discarded PDHM (Table 1).

**Table 1:** Comparison of PDHM utilization under the routine 24-h post-thaw usage policy and the simulated 48-h post-thaw usage policy. Because PDHM was supplied in standardized bottles of 30, 80, and 150 mL and OMM was used when available, the thawed PDHM volume did not always correspond to the administered PDHM volume. Under the simulated 48-h policy, the remaining thawed PDHM after feeding was assumed to be carried over for use on the following day

Infant	GA(weeks)	BW(g)	Thawed PDHM volume		PDHM volume used (mL)	Discarded PDHM volume		Saved PDHM volume (mL)
			24-h policy (mL)	48-h policy (mL)		24-h policy (mL)	48-h policy (mL)	
1	22 + 2	490	60	30	17	36	6	30
2	23 + 0	540	300	210	128	123	33	90
3	23 + 0	507	240	150	99	124	34	90
4	23 + 5	731	150	90	62	85	25	60
5	24 + 2	611	60	60	11	20	20	0
6	25 + 0	739	380	300	236	119	39	80
7	26 + 5	792	950	800	603	166	16	150
8	27 + 0	755	60	30	6	36	6	30
9	27 + 1	744	520	420	242	125	25	100
10	27 + 1	993	640	510	369	135	4	131
11	27 + 2	566	60	60	13	28	28	0
12	27 + 4	1053	1590	1500	1377	103	13	90
13	28 + 0	970	1260	1090	993	172	2	170
14	29 + 2	1063	90	60	13	56	12	44
15	29 + 5	1118	410	390	281	35	15	20
16	30 + 2	1393	1840	1750	1521	103	13	90
17	30 + 6	1131	2210	2090	1585	138	18	120
18	32 + 1	1367	2230	2010	1840	216	0	216
19	32 + 1	1423	2370	2240	2053	142	12	130
20	32 + 1	1695	2890	2760	2510	145	15	130
21	33 + 4	1450	2760	2600	2265	165	5	160
22	33 + 6	1406	250	210	97	58	18	40
23	34 + 4	1369	1160	1080	832	89	9	80
**Total**			**22,480**	**20,440**	**17,153**	**2419**	**368**	**2051**

## Discussion

Previous laboratory-based evaluations conducted in a human milk bank facility detected no bacterial growth in PDHM stored under refrigerated conditions for up to 48 h after thawing [9].

In the current study, conducted under routine NICU clinical conditions including standard handling and portioning procedures, no bacterial growth was detected up to 48 h after thawing. These findings not only support a previous laboratory-based study demonstrating the microbiological safety of PDHM during refrigerated storage, but also suggest the practical feasibility of implementing a 48-h post-thaw usage policy in routine NICU practice.

In Japan, PDHM is pasteurized using the Holder method (62.5 °C for 30 min), which minimizes infectious risks as much as possible. However, certain bacteria, such as *Bacillus cereus*, are not fully inactivated by this process [10].

Because PDHM distributed by milk banks undergoes strict screening and microbiological safety procedures, international guidelines have gradually shifted toward permitting longer post-thaw usage periods for PDHM under refrigerated conditions. Both HMBANA and more recent European evidence-based guidelines have revised their recommendations to permit the use of thawed PDHM for up to 48 h under refrigerated storage conditions [8, 11]. This revision has been supported by laboratory-based studies, particularly the study by Vickers et al., which demonstrated maintained microbiological purity of PDHM stored at 4 °C for up to 9 days [12]. Furthermore, a systematic review by Schlotterer et al. reported that three studies found no bacterial growth during 4–9 days of refrigerated storage after thawing [13].

If PDHM use is extended to 48 h post-thawing, careful attention is required when handling PDHM stored for beyond 24 h after thawing. Currently, PDHM exceeding 24 h is discarded; thus, without revising storage workflows, there is a risk that milk stored for 24–48 h may be inadvertently discarded or that milk exceeding 48 h may be mistakenly used. As routine portioning assumes that PDHM will be used within 24 h of thawing, modifications to labeling and storage procedures are essential. Confusion may arise when confirming lot numbers and determining the number of hours that have elapsed since the thawing. If a 48-h policy is adopted, comprehensive education and the establishment of clear, institution-specific storage protocols are necessary to prevent operational errors.

In Japan, PDHM is distributed in standardized bottles of 30, 80, and 150 mL with the primary aim of minimizing PDHM wastage.

During the first days after birth, when enteral feeding volumes are very small, the 30-mL bottles are used most frequently. As feeding volumes increase, or in NICUs where PDHM is administered to multiple infants simultaneously, the 80- and 150-mL bottles have been used more commonly because the contents can be distributed efficiently among several infants, thereby reducing waste.

However, because bottle sizes remain fixed while prescribed feeding volumes vary considerably, unused milk often remains after feeding and must be discarded under a 24-h post-thaw usage policy.

Bottle sizes differ considerably among countries. In many overseas human milk banks, the milk bank is located within the hospital. In addition, because milk bank operations are often performed by a limited number of staff, minimizing the handling required for repackaging is an important practical consideration. Consequently, some milk banks, particularly those using the Sterifeed system, deliver pasteurized PDHM to the NICU in the original 250-mL Sterifeed bottle without subdividing it into smaller containers.

Therefore, the magnitude of waste reduction achieved by extending the allowable post-thaw usage period may vary depending on bottle size and distribution practices. These differences should be considered when interpreting and generalizing our findings.

When enteral feeding is initiated in extremely low birth weight infants weighing < 1000 g, the typical starting volume is 1–2 mL per feed, administered every 3 h (eight feeds per day), resulting in a daily total of 8–16 mL. Consequently, even the smallest bottle resulted in approximately 20 mL waste. Under this feeding strategy, enteral feeding is commonly advanced by 20 mL/kg/day, doubling the required volume on the next day to 16–32 mL. When 32 mL is needed, an 80 mL bottle must be thawed, leaving 48 mL to be discarded. In contrast, if PDHM could be used for 48 h after thawing, the remaining 20 mL from the first day could be combined with an additional 30 mL bottle to provide an adequate supply. As demonstrated in this study, adopting a 48-h usage window substantially reduced waste volume.

This study has several limitations. It was conducted at a single facility, and the thawing and storage procedures varied between institutions.

From a practical perspective, the actual volume of PDHM waste may vary depending on feeding workflows and milk-handling protocols across NICU. In some facilities, PDHM bottles may be shared among multiple infants, potentially resulting in lower waste volumes than estimated in the present study. Conversely, in the NICU where mother’s own milk is frequently expressed at the bedside and used immediately, previously warmed PDHM may become unnecessary and require disposal despite remaining within the allowable post-thaw storage period. These findings highlight the importance of considering local clinical workflows and PDHM management practices when implementing extended post-thaw usage policies.

In summary, although not all PDHM can be used under all circumstances, extending the allowable post-thaw usage period to 48 h may increase the amount of PDHM that can be safely utilized while reducing unnecessary waste of this limited medical resource.

Ensuring a more stable supply of PDHM may help reduce complications during NICU hospitalization in preterm infants and support prolonged human milk feeding. Reducing unnecessary waste may also improve resource utilization and decrease the economic burden associated with donor milk processing and distribution. Operationally, extending the allowable usage period may reduce the frequency of thawing and milk portioning procedures, thereby improving workflow efficiency among NICU staff. Importantly, no bacterial growth was observed in the present study despite routine NICU handling and portioning procedures, supporting the microbiological feasibility of this approach in real-world clinical practice.

These findings complement existing evidence from North America and Europe and may contribute to the global harmonization of PDHM-handling practices.

## Conclusion

Thawed PDHM remained microbiologically sterile for up to 48 h under routine NICU conditions. Extending the allowable use period to 48 h could meaningfully reduce waste and promote more efficient and sustainable use of PDHM.

Efficient utilization of PDHM may also help ensure a stable supply of donor milk in the NICU, enabling the early initiation of human milk feeding and supporting breastfeeding. 

## Data Availability

All data supporting the findings of this study are included in this article.

## Abbreviations

PDHM: Pasteurized Donor Human Milk
NICU: Neonatal Intensive Care Unit
HMBANA: Human Milk Banking Association of North America
EMBA: European Milk Bank Association
CFU: Colony-Forming Units
